# Defining and Conceptualizing Technology-Facilitated Abuse (“Tech Abuse”): Findings of a Global Delphi Study

**DOI:** 10.1177/08862605241310465

**Published:** 2025-01-18

**Authors:** Nikolaos Koukopoulos, Madeleine Janickyj, Leonie Maria Tanczer

**Affiliations:** 1 University College London, UK

**Keywords:** technology- or image-based abuse, intimate partner violence/abuse, Internet and abuse, gender-based violence

## Abstract

Technology-facilitated abuse (TFA) describes the misuse or repurposing of digital systems to harass, coerce, or abuse. It is a global problem involving both existing and emerging technologies. Despite significant work across research, policy, and practice to understand the issue, the field operates within linguistic, conceptual, and disciplinary silos, inhibiting collaboration. To address this, the present study used the Delphi technique to reach a consensus on TFA conceptualization, definition, terminology, and measurement among subject experts. Following a literature review, a global, cross-disciplinary sample of academics, practitioners, and policymakers (*n* = 316) reflected on TFA across three survey rounds. The results showed both aligned and opposing perspectives. “Technology” and “facilitated” were the most preferable terms. Still, there was uncertainty regarding the need for additional terminologies to denote the scope of abuse, such as gendered descriptors. Participants had little familiarity with existing TFA measurement tools, with two-thirds unaware of any. Most experts agreed on conceptualizing TFA based on the perpetrator’s behavior, the victim’s harm and impact, and consent. They also supported an expansive TFA definition, beyond intimate relationships, that can involve groups and communities as perpetrators or targets. However, they were more reluctant to perceive TFA as a distinct abuse form, or one guided by social norms, legal thresholds, or involving child perpetrators. The findings are discussed in the context of the current TFA landscape, along with study limitations and steps to achieve a more unified TFA understanding.

The rapid development of digital systems has benefited modern societies but also created opportunities for the proliferation of harms. Specifically, the term “technology-facilitated abuse” (TFA) describes digitally enabled behavioral patterns involving harassment, abuse, and violence (Bailey et al., 2021). TFA is regularly discussed in the context of domestic abuse, where it is perpetrated via a range of systems, including personal electronic devices (phones, laptops, and tablets), smart home/Internet of things appliances, as well as online accounts, that are either shared or accessed without the partner’s consent (Tanczer et al., 2021). In the United Kingdom, 32% of women and children who sought support for domestic abuse in 2022 to 2023 (Refuge, 2023) and one in two adults in Australia experienced at least one form of TFA during their lives (Flynn et al., 2023).

The concept of TFA is often extended to include TFA carried out outside intimate partner domains. For example, in the definition provided by UN Women & the World Health Organization (2023) and adopted by the Global Partnership for Action on Gender-Based Online Harassment and Abuse, the terms “technology-facilitated gender-based violence (TFGBV)” and “technology-facilitated violence against women (TF VAW)” are employed to describe any act perpetrated through digital technology which leads to “physical, sexual, psychological, social, political, or economic harm, or infringements of rights and freedoms” (Global Partnership, 2023, p. 16). This broader conceptualization of TFA would consequently involve phenomena and behaviors such as hate speech (Paz et al., 2020), cyberbullying (Brochado et al., 2017), or violent extremism (Montasari, 2024). UN Women & the World Health Organization (2023) comment that a narrower conceptualization may facilitate comparisons but that a wider framework is needed to consider all relevant forms of technology’s adverse effects.

Alongside competing definitions, plenty of terms describe TFAs manifold subtypes and facets. These include, for example, “technology-facilitated intimate partner violence” (Mitchell et al., 2022), “technology-enabled financial abuse” (PenzeyMoog & Slakoff, 2021), and “technology-facilitated sexual violence” (Powell, 2022). In addition, terms frequently denote more specific phenomena that partially overlap with the concepts above, such as “image-based abuse” (Powell & Henry, 2017), “cyberviolence” (Peterson & Densley, 2017), or “online harassment” (Schoenebeck et al., 2023). Occasionally, there is a mismatch between the specificity of the term used and the behaviors described (Henry et al., 2020). For instance, Gámez-Guadix et al. (2015) utilized “online victimization” to refer to the “pressure to obtain unwanted sexual cooperation or the dissemination of a victim’s sexual content through the Internet” (p. 145) when, in reality, this description only accounts for sextortion, and (parts of) image-based sexual abuse.

Although terminological choices reflect particular languages, cultures, and jurisdictions (e.g., “technology-facilitated domestic and family violence” in Australia, “cyberviolences conjugales” in France) or areas of research, subject disciplines, and practices (e.g., smart home abuse), they may also signify an author’s theoretical approach or positionality. For instance, Dragiewicz et al. (2018) opted for the term “technology-facilitated coercive control” as they saw TFA as situated within a context of coercive and controlling behaviors, while “technology-facilitated domestic abuse” is commonly used in the intimate violence space (Douglas et al., 2019). Equally, the European Institute for Gender Equality (2017) adopted “cyber violence against women and girls,” given that their remit is violence against women and girls.

Due to the above variations and inconsistencies, the research field lacks comprehensive and standardized measurement tools (Brown & Hegarty, 2021). In 2022, the UN Secretary-General emphasized that the absence of agreed definitions and measures impedes any efforts to understand the true scale of TFA (United Nations General Assembly, 2022). Even though forms of TFA are evaluated through national surveys, these are only carried out in a limited number of countries, and certain forms of TFA are being omitted (University of Melbourne-UNFPA, 2023). Equally, various existing questionnaires capture only some aspects of TFA, such as the Technology-Facilitated Abuse in Relationships Scale (TAR; Brown & Hegarty, 2021), the Cyber-Dating Abuse Questionnaire (CDAQ; Borrajo et al., 2015), or the Cyber Psychological Abuse Scale (Leisring & Giumetti, 2014).

Limited but impactful research on TFA in countries of the Global South has been undertaken by organizations like the Association for Progressive Communications (2015), InternetLab (e.g., Neris et al., 2018), the Internet Democracy Project (e.g., Radhakrishnan, 2020), Pollicy (2020), and the Feminist Internet Research Network (e.g., McLean & Cicero, 2023). Even so, much of the current understanding of TFA is based on research in a few countries of the Global North and there is sparse knowledge of the nature and impact of TFA in communities facing multiple inequalities (IREX, 2023). For example, Bansal et al. (2024) highlighted the absence of evidence from Central Asia and the Pacific Islands, Glitch (2023) pointed out the difficulties in illustrating the extent of digital misogyny against Black women, and Brookfield et al. (2023) discussed the added burden of TFA for women with insecure immigration status.

The above-outlined challenges in conceptualizing, defining, and measuring TFA detrimentally impact the effective interaction among researchers, practitioners, and policymakers and the building of coordinated responses. To this end, this article presents the findings of a Delphi study involving a global and diverse pool of subject experts to expose converging and diverging points and unearth a common ground. The driving research questions for this study were:

RQ1: What is the most appropriate terminology, including non-English language terms? RQ2: How can TFA be defined and conceptualized? RQ3: How can TFA be measured? RQ4: What behaviors or patterns “count” as TFA? RQ5: What populations or groups is TFA relevant to? Before commencing the study, the researchers obtained ethics approval from the university’s Institutional Review Board.

## Method

The Delphi method is a structured process for achieving consensus among individuals with expertise on a particular topic (Jones & Hunter, 1995). It is an iterative process whereby the input from each survey round informs the next, with the degree of consensus being assessed at each point (Toma & Picioreanu, 2016). This process enables the participants to reflect on their opinions and allows for the modification of views (Hsu & Sandford, 2007).

**Figure 1.**
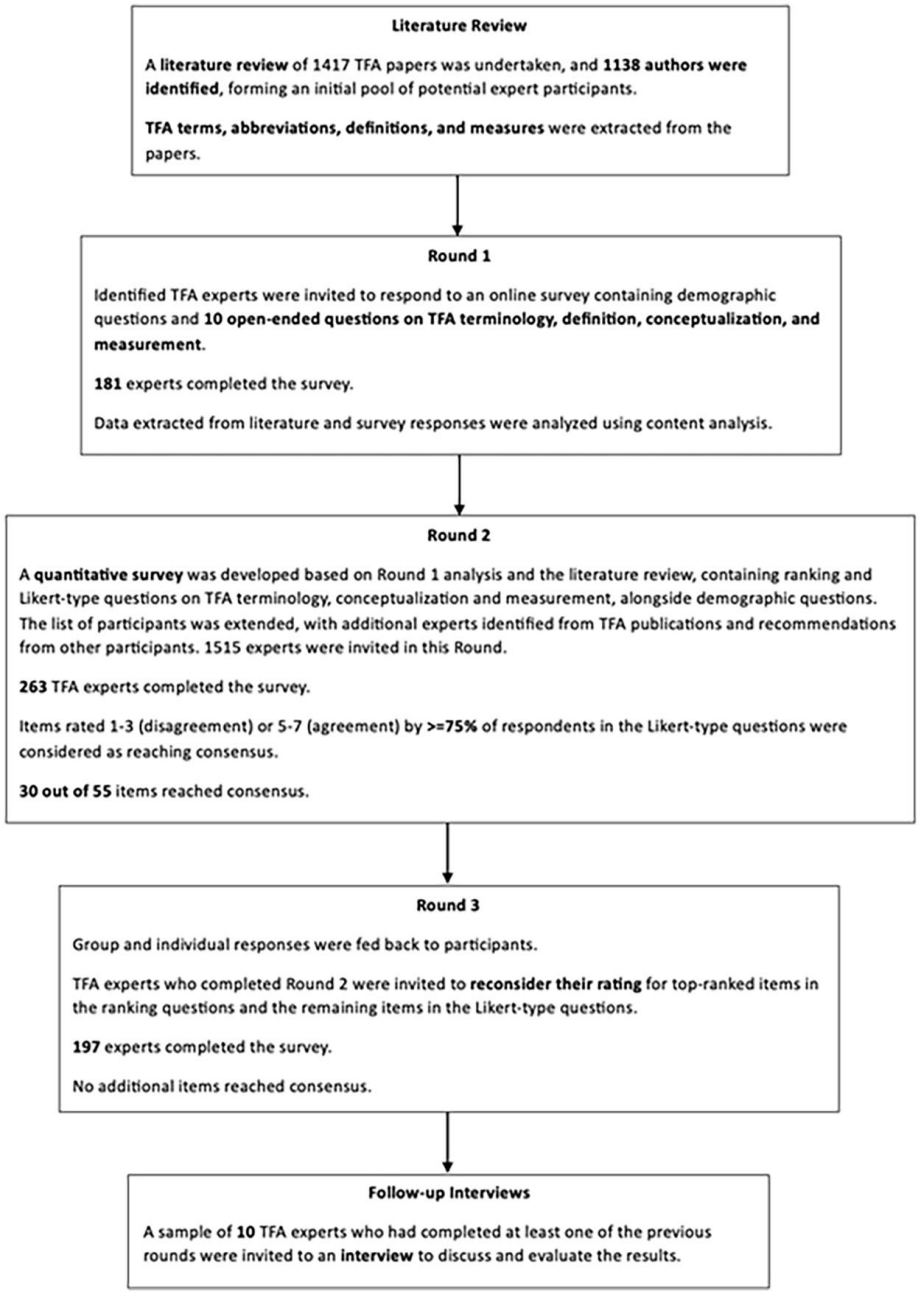
Illustration of the process.

The present study was completed over three rounds, with an additional interview follow-up to seek verbal feedback on the research and validate the results. An initial literature review on TFA was undertaken using Google Scholar (Supplemental Appendix A) using English language terms to (a) assist in the identification of suitable experts; (b) gather TFA definitions, terms, abbreviations, and measurements; and (c) inform the content of Round 1 (Fleuren, 2004; Stanyon et al., 2017). In total, 1,417 TFA papers were found, containing peer-reviewed publications (including systematic and scoping reviews), seminal books, and book chapters, as well as reports and policy documents (Figure 1). The papers were scanned, and the names of those who had authored or co-authored at least one publication or other relevant outputs were extracted (Supplemental Appendix B). Non-English language literature identified was also included. This was followed by the Round 1 survey, which consisted of 10 demographic and 10 open-ended questions (Supplemental Appendix C).

Round 2 was informed by TFA elements identified in the literature and responses provided by the expert participants in Round 1. It included demographic and multiple-choice questions in two formats: ranking and Likert-type questions. The ranking questions prompted the participants to order TFA terminology’s most favored or preferable elements. An additional “other” option was included, which could be populated with custom-made responses and rated accordingly, along with free-text boxes to allow for further elaboration. In the Likert-type questions, participants were asked to indicate their agreement on a 7-point Likert scale ranging from 1 (strongly disagree) to 7 (strongly agree). Each item also included a “don’t know” option, which participants were advised to choose should they not feel confident enough to provide a rating (Strafford et al., 2022; Vogel et al., 2019).

The Round 3 survey was like Round 2. However, it only included the top-ranked items of each ranking question and those that did not achieve consensus from the Likert-type questions. Participants were asked to re-rank/re-rate the items, after considering their personal and group ratings from Round 2. The follow-up interviews were semi-structured and drew on an interview guide (Supplemental Appendix D). All surveys and the follow-up interviews were in English.

### Participants

In the first round of the study, 1,138 TFA experts were invited, and 181 completed the survey (15.9% response rate). In the second round, 1,515 were invited, and 263 completed the survey (17.4%). In the third round, the 263 participants who had completed the second round were invited, and 197 completed it (74.9%). Together, a global, cross-disciplinary sample of academics, practitioners, and policymakers (*n* = 316) reflected on TFA across three survey rounds. Follow-up interviews were conducted with 10 participants which corresponded to the demographics of the overall sample surveyed. Table 1 presents the demographic characteristics of the expert participants across Rounds 1 and 2. Most identified as women (Round 1: 80.11%, Round 2: 78.54%) and were employed in Europe (Round 1: 44.75%, Round 2: 45.06%) and North America (Round 1: 33.15%, Round 2: 29.18%). Nonetheless, all continents/regions were represented in the sample.

**Table 1.** Participant Characteristics in Rounds 1 and 2.

Participant Demographics	Round 1 (*n* = 181)	Round 2 (*n* = 233)^a^
Gender		
Woman	145 (80.11%)	183 (78.54%)
Man	29 (16.02%)	45 (19.31%)
Non-binary	4 (2.21%)	3 (1.29%)
Prefer to self-describe	2 (1.10%)	2 (0.86%)
Prefer not to say	1 (0.55%)	—
Region of employment		
Africa	5 (2.76%)	15 (6.44%)
Asia	8 (4.42%)	9 (3.86%)
Europe	81 (44.75%)	105 (45.06%)
Latin America and the Caribbean	1 (0.55%)	2 (0.86%)
North America	60 (33.15%)	68 (29.18%)
Oceania	26 (14.36%)	34 (14.59%)
Level of expertise (self-assessed)		
Expert	44 (24.31%)	54 (23.18%)
Proficient	81 (44.75%)	93 (39.91%)
Competent	45 (24.86%)	61 (26.18%)
Advanced	10 (5.52%)	16 (6.87%)
Novice	1 (0.55%)	7 (3.00%)
Did not provide	—	2 (0.86%)
Area of work		
Academia	132 (72.93%)	140 (60.09%)
Nongovernmental organization	26 (14.36%)	32 (13.73%)
Governmental organization	9 (4.97%)	24 (10.30%)
Intergovernmental organization	—	14 (6.00%)
Private sector	6 (3.31%)	11 (4.72%)
Other	8 (4.42%)	12 (5.15%)
Academic discipline (if academic)		
Social work	8 (4.42%)	6 (2.58%)
Psychology	27 (14.92%)	24 (10.30%)
Criminology	19 (10.50%)	21 (9.01%)
Other	14 (7.73%)	11 (4.72%)
Computer/information science and engineering (including Human-Computer Interaction)	16 (8.84%)	21 (9.01%)
Media studies (including communication)	10 (5.52%)	16 (6.87%)
Political science (including policy)	5 (2.76%)	5 (2.15%)
Law	12 (6.63%)	14 (6.00%)
Anthropology	2 (1.10%)	3 (1.29%)
Economics	1 (0.55%)	2 (0.86%)
Philosophy	1 (0.55%)	1 (0.43%)
Sociology	12 (6.63%)	9 (3.86%)
Medicine and health	5 (2.76%)	7 (3.00%)

*Note.* This table demonstrates the demographic characteristics of Round 1 and Round 2 participants.

a The table shows the characteristics of the 233 participants who chose to provide their demographics in Round 2. In total, 263 participated in this round.

Most participants considered themselves competent, proficient, or expert on TFA (93.92%, 89.27%). Most worked in academia (72.93%, 60.09%), followed by those working in nongovernmental organizations (14.36%, 13.73%). Psychology, criminology, and computer science/engineering were the academic disciplines most frequently encountered in the expert sample. However, at least another 10 disciplines were represented, including social work, political science, law, anthropology, economics, philosophy, sociology, medicine, and health.

### Data Collection

The surveys were administered via Qualtrics and remained open for about 2 to 3 weeks each time. Pseudo-anonymization provided anonymity in line with the Delphi methodology (Taylor, 2020). Participants who at any point indicated that they were unable/did not want to participate or did not consider themselves subject experts were eliminated from the study. For the follow-up interviews, the researchers sent invitation emails to a sample of experts who had completed at least one of the previous rounds.

### Analysis

The survey data was exported to NVivo and responses to open-ended questions from each round were analyzed using content analysis, with “word” as the recording unit (Robson & McCartan, 2016). Coding was manual and no automatic coding function was used. MS Excel was used to calculate descriptive statistics and consensus metrics. This included percentages for ranking questions and cumulative percentages, as well as the median and the interquartile range for Likert-type questions. Consensus in the study was set a priori to 75%, meaning that, to achieve consensus for each Likert-type item, at least 75% of the participants would have to rate it with a 5 to 7 (somewhat agree, agree, strongly agree) or 1 to 3 (somewhat disagree, disagree, strongly disagree). The anonymized datasets associated with this study are available at the university’s research data repository (Koukopoulos et al., 2024).

## Results

The analyzed publications and Delphi responses revealed clustered perspectives on TFA, uncovering both united viewpoints and fragmentation in the field. In the literature review and Round 1, a wide range of terminologies, definitions, conceptualizations, and measurements were collated and further agreed upon in Rounds 2 and 3. Consensus was achieved for 30 out of the 55 Likert-type items. Participants ranked “technology” and “facilitated” as the most preferred elements in the TFA terminology. However, the use of a gendered descriptor (e.g., “gender-based” or “against women and girls”) was contested, and there was limited to no knowledge of relevant measurement tools. The participants offered a broad understanding of TFA, which included domestic abuse contexts but also extended to professional environments and cases where the perpetrator is unknown/a stranger to the victim-survivor. Participants would not exclude most groups from a conceptualization of TFA, but their opinions were less aligned regarding whether acts normally regarded as TFA are still understood as such when perpetrated by (prepubescent) children or against institutions (e.g., acts against an organization or individuals associated with a particular organization, such as a domestic abuse charity).

### Literature Review and Round 1

During the literature review, 149 TFA *terms* were identified, with many also mentioned by subject experts in Round 1. In the open-ended survey, participants referred to over 500 different terms. Some were “umbrella terms”—phenomena on the same conceptual level as TFA (e.g., TFGBV)—while others indicated conceptually specific abusive behaviors involving technology, which participants would place under TFA (e.g., doxxing). Overall, the most suggested terms included “technology-facilitated abuse,” “image-based sexual abuse,” “cyberstalking,” “revenge porn,” “tech abuse,” and “online harassment.” In addition, 12 non-English terms were found in the literature, and 144 in 19 different languages were named by participants, including “Digitale Gewalt” (German), “Ciberviolencia” (Spanish), and “Abuso Digital Nos Relacionamentos Afetivosexuais” (Brazilian Portuguese).

Eighty-one TFA *measurement tools* were sighted in the literature, and 66 were suggested by participants, with approximately 35% overlap between the measurements identified across the two sources. The most common instrument cited by participants included the TAR (Brown & Hegarty, 2021), the CDAQ (Borrajo et al., 2015), and the Digital Dating Abuse Scale (DDA; Reed et al., 2016).

When asked which groups would be *excluded* from the conceptualization of TFA, most participants (71.34%) responded that they would not discount any. This was consistent with the TFA literature, where different types of population groups were represented, such as children and adolescents (Rowse et al., 2022), older adults (Bayne et al., 2023), patients/those in a clinical or medical context (Straw & Tanczer, 2023), or minoritized communities (Glitch, 2023; Vogler et al., 2023). That said, certain population groups appeared more often in both the literature as well as the expert survey as victims-survivors or perpetrators of TFA, such as adults in a domestic abuse context. Children, however, were, on occasion viewed as a distinct group that should be separated from TFA conceptualizations due to differences in their culpability according to the law, or a distinct perception of their safeguarding and regulatory needs.

Many participants felt there should not be any conceptual boundaries or *thresholds* for what counts as TFA. Conversely, some experts highlighted that the victim-survivor’s perception of the abuse, the impact it had on them, the presence or absence of consent, and what is reasonable within a particular society and culture should be factored in when thinking about what does or does not fall within the TFA remit.

An analysis of the *definitions* collated from the literature and survey responses gave prominence to the following elements: “technology as a medium,” “role of technology in abuse,” “abusive acts/behaviors,” “technologies/apps/devices,” “social and societal context,” and “nature of harm.” These were considered aspects that should feature in a description of TFA and are elaborated on in more depth below.

When referring to the *medium* via which the harmful behavior is perpetrated, the most common term used was “technology” or “technological” (e.g., technological tools), followed by “digital.” Other terms less frequently mentioned included “online” and “Internet.” Various terms were utilized to describe the *role of technology* in the abuse. The most prevailing was the word “facilitated,” followed by “enabled,” “assisted,” “aggravated,” “amplified,” “mediated,” and “misused.” Several others were only mentioned once or twice, such as “exacerbated,” “dependent,” and “extended.”

In discussing *abusive acts or behaviors*, publications and participants referred to categories denoting a range of thematically similar conduct. The following were frequently stated: controlling behavior, stalking/monitoring/surveillance behavior, threatening behavior, coercive behavior, or violent behavior and harassment. Other behaviors were also specified, though less frequently: intimidation, defamation, impersonation, exploitation, humiliation, insults, isolation, manipulation, gaslighting, unwanted communication, or any behavior that violates consent. A few practices were only quoted once or twice, including gaslighting, unwanted contact, aggression, and deception. Broader categories included image-based abuse (including image-based sexual abuse, sextortion, and nonconsensual/coerced sexting), cyber-dating abuse, cyberbullying, computer/device hacking, trolling, and fraud.

Particular *technological tools* were repeatedly mentioned as a means by which TFA may be carried out. These included location or activity tracking, generative AI (e.g., deepfake images), and unauthorized/limited/nonconsensual access to digital products and services. A range of apps and online platforms that may be used for TFA were highlighted, including social media, email, spyware, messaging applications, and chatgroups. Dating apps, parental control apps, and online forums were also considered, although less often. Some analyzed publications and survey responses also referred to devices via which TFA may be performed, including smartphones, computers, smart home appliances, cameras, vehicles, standalone GPS tracking products, and surveillance tools.

The identified definitions also pointed toward the *social context* in which TFA acts occur. For example, whenever certain groups were flagged, these would frequently include intimate partners and dating relationships, with respondents alluding to the interpersonal or the “domestic” aspect of the abuse. However, several publications and expert participants acknowledged the importance of situating TFA across a spectrum and extending it to acquaintances, online encounters, colleagues, parents, children, or strangers. The earlier discussed “broader” understanding of TFA was hereby central to them. Others again highlighted the gendered dimension of the abuse, evidenced by phrases such as “gender-based,” “against women,” and “against women and girls.” Additional societal considerations featured, including the need to be mindful of existing societal power structures, the inclusion of all gender/sexual minorities, and the recognition of intersectionality, namely “the various ways in which gender and race intersect in shaping structural, political, and representational aspects of violence against women of color” (Crenshaw, 1991, p. 1244).

Finally, there were references to the *nature of harm* and the *perpetrator’s intent*. When examining the former, TFA was mainly understood as an extension of harm inflicted offline. An association was further made to physical, psychological, sexual, and emotional abuse. Economic, social, and political harm were mentioned less regularly, as were spiritual harm and infringement of rights. Although many definitions and survey responses did not discuss intent or argue that premeditation would not be necessary to define TFA, those who did remarked that the requirement of *causing harm or distress to another* was of utmost importance, followed by the need to *maintain power and control* and the necessity for the abusive actions to be *deliberate or intentional* as opposed to accidental.

### Round 2

Round 2 involved ranking exercises and close-ended, “tick-box” questions. In rating TFA terminological elements, the majority of participants endorsed the *prefix* “technology,” which was most often placed first (54.0%), with “digital” placed second (32.3%). However, “digital” appeared in the top half of the ranking board far more frequently (93.5%) than “technology” (84.4%), while “online” and “cyber” were also in the top half, with 67.3% and 71.5%, respectively. In considering a term to reflect the *role of technology* in the abuse, most participants chose “facilitated” first (57.4%) and “enabled” second (27.4%). “Facilitated” was also repeatedly appearing in the top half of the ranking board (92.4%), proceeded by “enabled” (85.2%), “assisted” (78.3%), and “based” (57.8%).

When prompted about how participants would rank terminology elements to denote the *scope of violence and abuse*, experts appeared divided. “Gender-based” was most often placed first but only by 33.1% of the sample, while “interpersonal” was placed first by 21.3%. Overall, 76.4% of the participants placed “gender-based” in the top half of the ranking board, followed by “against women and girls” (70.7%), “against women” (67.3%), “coercive control” (62.7%), “intimate partner” (55.9%) and “interpersonal” (52.9%). Finally, when encouraged to indicate their most preferred term overall, 17.3% of the participants opted for “technology-facilitated abuse,” with other terms suggested by a substantially lower proportion of respondents, such as “cyber abuse” (4.1%) and “digital violence” (2.2%).

Only about a third (32.8%) of participants reported awareness of TFA *measurement* frameworks. Two-thirds (66.3%) of those who indicated familiarity with some rated the TAR (Brown & Hegarty, 2021) as the most effective in capturing TFA phenomena. Other measures were also selected, though by fewer participants: the Technology-Facilitated Sexual Violence Victimization Scale (TSVV; Powell & Henry, 2019) (55.8%), the CDAQ (Borrajo et al., 2015) (37.2%), the Intimate Partner Cyber Abuse Questionnaire (Fissel et al., 2022) (34.9%), and the DDA (Reed et al., 2016) (26.7%).

**Figure 2.**
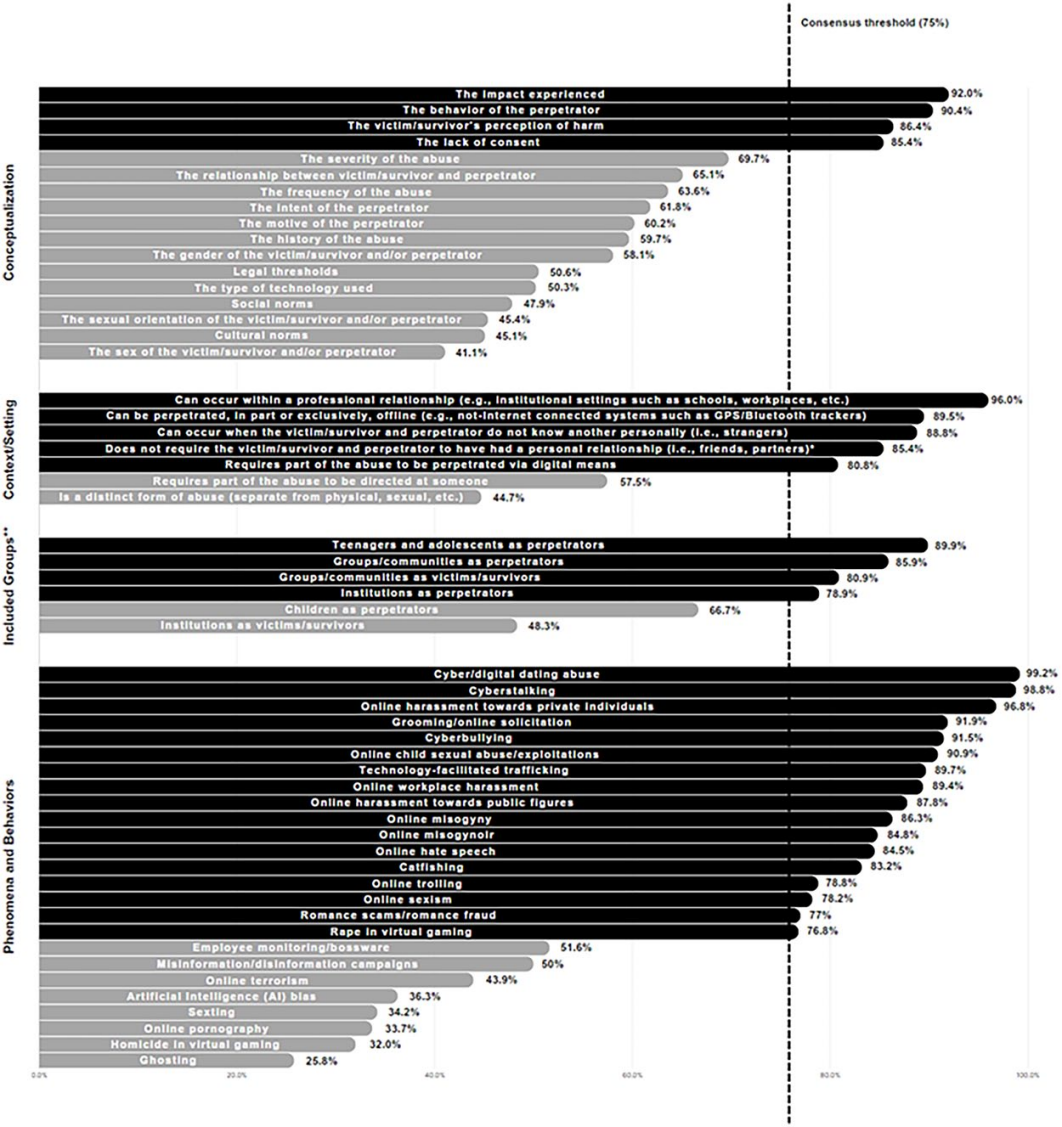
Level of expert agreement (in percentage) after Round 3. *Note.* This figure demonstrates the percentage of agreement for all Likert-type items after Rounds 2 and 3. Thirty items achieved consensus (black), whereas 25 did not (gray). *Negation was added to the phrasing of the original item as participants reached a consensus in the opposite direction (disagreed with the original statement). **The phrasing was modified from “Excluded Groups” to “Included Groups” as consensus was achieved at the opposite end of the scale (participants disagreed with the original statement).

When prompted to rate *elements that should guide TFA conceptualization*, four items reached consensus (Figure 2): “the impact experienced” (92%), “the behavior of the perpetrator” (90.4%), “the victim-survivor’s perception of harm” (86.4%), and “the lack of consent” (85.4%). Thirteen items did not reach consensus in Round 2 and were reposted in Round 3. In responding to the question of the *context or setting* in which TFA can occur, four items reached consensus: “can occur within a professional relationship” (96%), “can be perpetrated, in part or exclusively, offline” (89.5%), “can occur when the victim-survivor and perpetrator do not know one another personally” (88.8%), “requires part of the abuse to be perpetrated via digital means” (80.8%). An additional item reached consensus at the opposite end of the rating scale, with participants agreeing that it should *not* be a requirement for the “victim-survivor and perpetrator to have had a personal relationship” (85.4%). Two items did not reach consensus and were sent to Round 3.

When assessing whether certain groups should be *excluded* from the conceptualization of TFA, participants agreed that “teenagers and adolescents as perpetrators” (89.9%), “groups/communities as perpetrators” (85.9%), “groups/communities as victims-survivors” (80.9%), and “institutions as perpetrators” (78.9%) should not be excluded. Two items did not reach consensus and were presented to the participants again. In the question of *phenomena and behaviors* which fall under their conceptualization of TFA, 17 items achieved consensus: “cyber/digital dating abuse” (99.2%), “cyberstalking” (98.8%), “online harassment toward private individuals” (96.8%), “grooming/online solicitation” (91.9%), “online child sexual abuse/exploitation” (90.9%), “cyberbullying” (91.5%), “technology-facilitated trafficking” (89.7%), “online workplace harassment” (89.4%), “online harassment toward public figures” (87.8%), “online misogyny” (86.3%), “online misogynoir” (84.8%), “online hate speech” (84.5%), “catfishing” (83.2%), “online trolling” (78.8%), “online sexism” (78.2%), “romance scams/romance fraud” (77%), and “rape in virtual gaming” (76.8%). Eight items, however, did not achieve consensus and were reconsidered by the experts in Round 3.

The responses of participants from different demographic backgrounds offer valuable insights into how their understanding of TFA varies. In the question regarding the scope of abuse, 28.6% of those from intergovernmental organizations placed “against women” and 14.3% placed “against women and girls” first (42.9% combined). Other sectors did not choose these two elements as frequently. At the same time, half of those from intergovernmental organizations placed “gender-based” first, while it was picked less often by academics (placed first by only 26.4%). A high percentage of the intergovernmental sector also agreed that gender (92.86%) and sexual orientation (85.7%) should guide our conceptualization of TFA. These elements were not as popular across the sample (66% and 55.4%, respectively). Yet only half (50%) of the intergovernmental sector agreed that the lack of consent should guide our TFA conceptualization, despite the overall agreement being 85.4%.

Examining the rating of items across disciplines also reveals a somewhat contrasting picture. 57.1% of Law and 50% of Media Studies academics placed “gender-based” first. However, the term did not prove as popular with Computer/Information Science (0%), Criminology (14.3%), and Psychology (16.7%) scholars. Equally, of those with a Law background, 92.9% agreed that “gender” and 78.6% agreed that “sexual orientation” should guide our TFA conceptualization. The rates were significantly lower for Computer/Information Science (52.4% and 47.6%, respectively), and Criminology (52.4% and 42.9%, respectively). “Misinformation/disinformation campaigns” were seen as TFA by Law (64.3%) and Media Studies (62.5%) scholars but not as many Computer/Information scientists (33.3%) and Psychologists (37.5%) agreed. Similar disparities were noted for “online child sexual abuse/exploitations” (66.7% agreement from Psychology vs. 90.9% agreement overall), “online misogyny” (61.9% in Computer/Information Science, 62.5% in Psychology vs. 92.9% in Law and 90.5% in Criminology), “online trolling” (47.6% in Computer/Information Science and 58.3% in Psychology vs. 87.5% in Media Studies, 85.7% in Law and 81% in Criminology), and “romance scams/fraud” (81% in Criminology vs. 50% in Psychology).

Differences were also noted according to the level of expertise of the participants. Despite “technology” being the by far most chosen term among experienced scholars, only 28.6% of novices placed it first, opting for “digital” instead (42.9%). Significantly more novices agreed on the importance of the “type of technology” used (100% vs. 57.8% overall), “legal thresholds” (71.4% vs. 45.9% overall), as well as “cultural” (85.7% vs. 50.4% overall), and “social norms” (85.7% vs. 55.97% overall) in TFA conceptualization. Novices were also more confident in placing “AI bias” (85.7% vs. 51.8% overall), “misinformation/disinformation campaigns” (100% vs. 62.1% overall), “online pornography” (71.4% vs. 37.4% overall), and “online terrorism” (85.7% vs. 58.6% overall) within TFA.

### Round 3 and Follow-Up Interviews

In Round 3, participants’ ranking and rating of items were congruent with Round 2 results. When presented with the top half of the *prefix* items from Round 2, the order was maintained: “technology” was placed first by most participants (49.2%), and “digital” was placed second by most (44.7%). “Cyber” primarily appeared in the third place (39.6%) while “online” came up fourth (47.2%). Equally, in the *role of technology* question, “facilitated” was most regularly placed first (81.2%), “enabled” was most often positioned second (54.31%), “assisted” was third (48.7%), and “based” fourth (60.9%) (Table 2).

**Table 2.** Overall Positions of the Terminology Elements in the Ranking Exercise (Round 3).

Prefix	Role of Tech	Scope of Violence
1. Technology (49.2%)	1. Facilitated (81.2%)	1. Gender-based (59.4%)
2. Digital (44.7%)	2. Enabled (54.3%)	2. Against women and girls (45.7%)
3. Cyber (39.6%)	3. Assisted (48.7%)	3. Against women (42.6%)
4. Online (47.2%)	4. Based (60.9%)	4. Coercive control (37.1%)
—	—	5. Intimate partner (39.6%)

*Note.* This table demonstrates the overall ranking of the terminology elements in the ranking exercise (Round 3) along with the percentage of participants who placed them in this position.

When commenting on their preferred terminology, some experts highlighted that “technology” as a prefix was broader and more flexible than others. At the same time, “digital” could be helpful when more specific terminology is needed. Others suggested that “facilitated” emphasizes the perpetrator rather than the technology, while “based” was seen positively and negatively. For example, some saw “based” as too limited, while others highlighted that it is more neutral and could be more easily translated into other languages.

When ranking the *scope of violence and abuse* items, “gender-based” usually appeared in the first place (59.4%) and “against women and girls” in the second (45.7%), followed by “against women,” which was mostly placed third (42.6%). Finally, “coercive control” and “intimate partner” were most often categorized fourth and fifth, with 37.1% and 39.6%, respectively. When reasoning their choices, some participants supported the use of a gender descriptor, while others felt that this could limit the scope of the definition and exclude populations who may be marginalized because of characteristics other than gender (race, sexual orientation, disabilities, etc.). Some indicated their preference for “gender-based” as opposed to “against women” or “against women and girls.” The latter two could imply a disregard for TFA victims-survivors who identify as men or non-binary.

Across the Likert-type questions, no additional items reached the consensus threshold (Figure 2). In terms of *elements that should guide TFA conceptualization*, the ratings across the 13 items were: “the relationship between the victim-survivor and perpetrator” (65.1%), “the motive of the perpetrator” (60.2%), “the intent of the perpetrator” (61.8%), “the severity of the abuse” (69.73%), “the frequency of the abuse” (63.6%), “the gender of the victim-survivor and/or perpetrator” (58.1%), “the history of the abuse” (59.7%), “the type of technology used” (50.3%), “social norms” (47.9%), “the sexual orientation of the victim-survivor and/or perpetrator” (45.4%), “cultural norms” (45.1%), “legal thresholds” (50.6%), and “the sex of the victim-survivor and perpetrator” (41.1%).

In considering the *context or setting* of the abuse, the ratings were: “requires the abuse to be directed at someone” (57.5%), and “is a distinct form of abuse” (44.7%). In whether certain groups should be *excluded* from the conceptualization, “children as perpetrators” (66.7%) and “institutions as victims-survivors” (48.3%) did not reach the consensus threshold. Finally, the eight items of TFA *phenomena and behaviors* carried over from Round 2, also did not reach consensus (Figure 2): “sexting” (34.2%), “homicide in virtual gaming” (32%), “online pornography” (33.7%), “artificial intelligence (AI) bias” (36.3%), “employee monitoring/bossware” (51.6%), “ghosting” (25.8%), “misinformation/disinformation campaigns” (50%), and “online terrorism” (43.9%).

In the follow-up interviews, participants were overall positive about the aims and usefulness of the study. However, some thought agreeing on a common TFA definition or terminology may be too ambitious or unattainable. Others commented that an “onion-like” conceptualization, involving a broader definition followed by several narrower and context-specific ones, may be more effective. Although the interviewees acknowledged that TFA disproportionately affects women, they appeared divided regarding the inclusion of a gendered descriptor; some supported it, but others saw it as too restrictive, or running the risk of missing out on other groups (e.g., the LGBTQI+ community).

More generally, subject experts highlighted the importance of considering cultural differences and protected characteristics and emphasized variations in how these are perceived across countries, communities, and cultures. When discussing whether children as perpetrators should be included in a TFA definition, most interviewees distinguished between harm and liability: TFA perpetrated by children is harmful behavior and therefore should be included, even if the actors are not considered legally liable due to their age.

## Discussion

The results of this global Delphi study indicate that subject experts widely share viewpoints about how to conceptualize, define, and measure TFA, yet some elements remain contested. Overall, participants agreed that TFA includes intimate and professional relationships, but can be perpetrated against strangers. Although respondents acknowledged the requirement of at least part of the abuse to be perpetrated via digital means, they also endorsed that TFA can be committed partially or exclusively offline/without Internet-enabled devices. The impact experienced, the behavior of the perpetrator, the victim-survivor’s perception of harm, and lack of consent, were all important elements of TFA conceptualization. The majority agreed to account for most population groups to be subsumed within a TFA definition. Still, experts appeared uncertain on whether acts normally perceived as TFA would still be regarded as TFA when committed by (prepubescent) children or against institutions. The preferred elements to describe TFA were “technology” (with “digital” a close second) and “facilitated,” while “gender-based” was picked repeatedly to define the scope of violence and abuse. “Technology-facilitated abuse” was also the most preferred term overall.

This expansive understanding of TFA uncovered and shared among participants aligns with present conceptualizations from international stakeholders, such as the Global Partnership (2023) and UN Women (2023). The elements, phenomena, and behaviors agreed upon in the study further indicate TFA’s “conceptual boundaries.” As expected, the behavior of the perpetrator, its impact, and the harm inflicted on the victim-survivor were seen as central in characterizing TFA. Indeed, TFA has been shown to affect victim-survivors significantly (Rogers et al., 2023), prolonging and intensifying the effects of non-technological abuse (Fiolet et al., 2021), overcoming physical boundaries and, thus, creating “spaceless” ways of causing harm (Harris, 2018). The surveyed subject specialists also highlighted the importance of consent in TFA conceptualization. While consent is included in some TFA definitions, it is more explicitly addressed in certain TFA domains, such as image-based sexual abuse (Patel & Roesch, 2020).

The use of the broader term “technology” over more specific options like “digital” or “online” was to be expected given that it appears to be more encompassing, and more publications to date refer to it. A study by Thinks Insights (2024), commissioned by the U.K.’s National Cyber Security Center, highlighted that the public and young people, in particular, more often associate “technology” with progress, advancement, and innovations (such as AI), whereas “digital” is now deemed to be obsolete, as a lot of devices are now “smart.” Our findings consequently suggest that “technology” is perhaps a more “future-proof” term.

Most experts recognized disciplinary and linguistic silos within the TFA field. This is evident in the disparities between how different sectors (e.g., academic, nongovernmental, and governmental/intergovernmental) use and endorse terminology around gendered and sexual orientation descriptors, even as the core concept of TFA is similarly understood. For example, governmental/intergovernmental organizations such as the U.K. Government (HM Government, 2021), UNFPA (2021), UN Women (2023), and the Global Partnership (2023) use different terminology to what is found in the academic literature on the topic.

The question of whether perpetrators’ intent should be part of the conceptualization did not reach a consensus, mirroring criticisms of TFA-related legislation. For example, in the United Kingdom, the perpetrator’s intent to cause distress has been difficult to prove in cases of image-based sexual abuse (Huber, 2023; McGlynn, 2022), leading to the removal of that requirement from the Online Safety Act (2023). Nevertheless, the “intent” criterion is still included or considered in other legislative efforts, such as in the new cyberflashing legislation (also part of the above Act) and proposed legislation on deepfake images in the U.K. Criminal Justice Bill (Ministry of Justice, 2024). Together, this suggests an ongoing debate around the role of intent in TFA-related laws and policies.

The inclusion of children as perpetrators of TFA remains a point of contention, even as adolescents are more commonly recognized. While the perpetration of TFA by teenagers or young adults is often viewed as TFA (e.g., digital dating abuse; Afrouz & Vassos, 2024), there is greater reluctance to categorize the same behaviors enacted by young children in this way, even if some of the corresponding phenomena are (i.e., cyberbullying). In their comments, some participants mentioned the risks of considering children criminally liable if included as perpetrators. Others commented on the fact that there is simply not enough research on this topic or that younger children have less access to technology. However, research indicates a growing exposure to and engagement with technology by very young children, particularly touchscreen devices (Mantilla & Edwards, 2019; Pew Research Center, 2020). A preference for more age-appropriate terminology when discussing TFA among minors (e.g., technology-assisted harmful sexual behavior; Lewis, 2018) could be an additional reason explaining the hesitancy to include children as perpetrators.

The inclusion of “sexting” and “online pornography” also lacked consensus among participants. This may be because both can be viewed as either consensual/positive, or nonconsensual/harmful (Raine et al., 2020; Schokkenbroek et al., 2023), which would also explain the relatively high number of “don’t know” responses. Similarly, “Artificial Intelligence (AI) bias” did not reach a consensus and had high percentages of “don’t know” responses. Possible explanations could be that AI bias is not directly tied to the intentions of a specific perpetrator or due to a limited understanding of its potential implications for TFA, such as in the case of the reinforcement of bias and gender-based violence by Large Language Models (UNESCO & IRCAI, 2024).

Although the initial literature review identified several scales and questionnaires measuring some aspects of TFA, most expert participants were unfamiliar with them. Given the limited quantitative research in this field, this lack of awareness is not entirely surprising and is a gap previously highlighted by scholars (Bailey et al., 2021). Moving forward, the existing measurement tools could serve as a starting point for developing a more comprehensive TFA assessment. In addition, accessing and analyzing secondary/administrative data could help illustrate the scale of the problem across different countries, communities, and cultures. Given the acknowledged need for a validated, wide-ranging TFA measure to collate representative data (University of Melbourne-UNFPA, 2023), these approaches may help advance research and understanding in this emerging field.

The lack of consensus on whether to include gender, sexual orientation, and social and cultural norms in TFA conceptualization does not necessarily signify their lack of importance. Rather, it may reflect concerns that incorporating such specific factors could narrow the definition’s scope and reduce universality. Nevertheless, it can also be argued that highlighting these issues can help emphasize the significant burden of TFA on particular groups, while still recognizing its broader effect on communities with different characteristics. For example, the Council of Europe (n.d.) uses the term “gender-based violence,” acknowledging that it disproportionately affects women but that it is also applicable to LGBTQI+ individuals and men. In addition, as seen in the context of gender-specific terminologies, there appear to be notable differences in how these concepts are approached across sectors, such as between intergovernmental and academic domains.

A key limitation of this study is that it only provides a “snapshot” of TFA, given the constantly evolving nature of the phenomenon. Hence, the findings primarily reflect the current understanding of the expert participants sampled. Moreover, while efforts were made to recruit a globally diverse set of participants, the consensus was largely based on experts from major communities in the Global North. It is also notable that less than a fifth of the expert participants were men. Although this may in part reflect gender representation in the wider field, it remains critical to engage men in the prevention of gender-based violence (Peacock & Barker, 2014). Another limitation is that some areas initially deemed out of scope, such as cyberbullying and online child sexual abuse/exploitation, were later included after some participants viewed them as relevant to TFA. This reflects the challenges in delineating the conceptual boundaries of this emerging field. Finally, the study acknowledges that not all TFA behaviors were included in the questionnaires, as the focus was on understanding the core conceptual parameters rather than an exhaustive catalog.

The fragmentation and contrasting conceptualizations of TFA observed in this research underscore the need for greater cross-disciplinary communication among researchers, practitioners, and policymakers to move closer toward a unified understanding of TFA. Some form of standardization is particularly crucial, given the rapidly developing ways existing and emerging technologies are weaponized in the digital realm. Concrete, practical steps could help bridge these divides by consolidating published work into a searchable database. This could include suggestions for conceptually similar terminology across various sectors and subject areas. Furthermore, an interactive online map of key TFA stakeholders and research groups could facilitate greater collaboration and knowledge-sharing, which our research team is now working on.

## Supplemental Material

sj-docx-1-jiv-10.1177_08862605241310465 – Supplemental material for Defining and Conceptualizing Technology-Facilitated Abuse (“Tech Abuse”): Findings of a Global Delphi Study Supplemental material, sj-docx-1-jiv-10.1177_08862605241310465 for Defining and Conceptualizing Technology-Facilitated Abuse (“Tech Abuse”): Findings of a Global Delphi Study by Nikolaos Koukopoulos, Madeleine Janickyj and Leonie Maria Tanczer in Journal of Interpersonal Violence
