# Supplementary material for: Defining and Conceptualizing Technology-Facilitated Abuse (“Tech Abuse”): Findings of a Global Delphi Study
Source: J Interpers Violence. 2025 Jan 18;41(1-2):249–75. doi: 10.1177/08862605241310465 (PMC12662827; doi:10.1177/08862605241310465)
Supplement: sj-docx-1-jiv-10.1177_08862605241310465 – Supplemental material for Defining and Conceptualizing Technology-Facilitated Abuse (“Tech Abuse”): Findings of a Global Delphi Study [file sj-docx-1-jiv-10.1177_08862605241310465.docx]

**Appendix A. Literature review search terms**

**The search terms used in Google Scholar searches were:**

“technology_facilitated_violence”, “cyber_violence”, “digital_dating_abuse”, “online_violence”, “tech_facilitated violence”, “technology_facilitated abuse”, “cyber_abuse”, “online_gender_based_violence”, “technology_facilitated_sexual_violence”, “doxing”, “doxxing”, “image_based_sexual_abuse”, “cyber-sexual violence”, and “image_based_sexual_abuse”.

**Appendix B. Expert inclusion criteria**

**The inclusion criteria for expert participants were:**

- Individuals who had authored or co-authored at least one publication or other relevant outputs falling within the following areas: online harassment, online violence, technology-facilitated gender-based violence, cyber dating violence, cyber dating abuse (CDA), cyber IPV, image-based abuse, intimate image abuse, image-based sexual abuse, revenge porn, dating app abuse, online abuse, spyware, stalkerware, spouseware, digital abuse, nonconsensual pornography, sexting, cyber sexual violence, technology-facilitated sexual violence, doxxing, doxing, digital gender-sexual violations, deepfakes, cyberabuse, online safety, online harm, cyberstalking.
- Additional contacts were added from other sources available to the researchers: (1) an advert placed in the research group’s monthly newsletter, (2) experts approached via relevant online networks, and (3) the researcher’s professional contacts, including related contacts from social media sites (i.e., Twitter/X).

**The exclusion criteria for expert participants were:**

- Those working on hate speech, child (sexual) abuse, and cyberbullying were excluded, as these thematic areas were considered out of the scope of the study. Their inclusion would have drastically expanded the literature search and future participant pool.
- Police officials/law enforcement personnel and support sector organizations with no known public trace of their TFA expertise were excluded. Only policy officials who had publicly available publications on the issue of TFA (e.g., United Nations officials) were included.

**Appendix C. Survey questions**

**Demographic questions (Round 1 and 2)**

Age, gender identity, area of work and disciplinary background, country of work, years of engagement with TFA, knowledge, and expertise.

**Round 1 Main questions**

1. Based on your knowledge and experience, how would you **define** **or describe** **tech abuse**? Feel free to elaborate as much or as little as you want in your response.
2. If you know one or several definitions of tech abuse (e.g., from the literature, legislation, an organization) **with which you can agree**, please cite these in the box below. Please include a reference, such as a hyperlink to the source.
3. In your view, should behaviors meet a **certain threshold** for them to "count" as tech abuse? For instance, how severe/frequent/technologically advanced/etc. do they need to be?
4. What **terms** (e.g., cyber IPV, ICT-facilitated violence, spouseware etc.) and **abbreviations** (e.g., TFDA, OGBV, IBSA etc.) have you heard/do you know are used to describe the phenomenon of tech abuse? Please refer to as many of them as possible without considering their prevalence or popularity.
5. From all available terms and abbreviations to describe the phenomenon of tech abuse, **which ones do you prefer** to use in your work, research, or practice? And why?
6. If you speak a **different language**, have you encountered non-English words describing a tech abuse phenomenon?
7. Are you familiar with any **metrics** to quantitatively measure tech abuse? Please refer to any survey instruments and scales (e.g., TAR scale, OSH scale) you encountered and feel free to insert hyperlinks to point us towards publications.
8. Do you exclude any **population groups** from your conceptualization of tech abuse (e.g., children, young adults)? How and why should we set such boundaries?
9. Based on your knowledge and experience, what would be the **best way to agree** on and popularize a unified/standardized terminology, definition, and measurement of tech abuse?
10. Do you have any final questions, points, comments, or concerns you would like to share with the research team.

**Round 2 Main questions**

1. To achieve consensus on the **most-favored prefix**, please rank the following terms according to your preference:

Computer. Cyber, Digital, Electronic, Information and Communication Technologies (ICT), Online, Technology, Other.

1. Please rank the following terms according to your preference to **describe technology’s role** in tech abuse:

Assisted, Based, Dependent, Enabled, Enacted, Facilitated, Mediated, Other.

1. Please rank the following terms according to your preference, to **describe the scope of violence/abuse** in tech abuse:

Against Women, Against Women and Girls, Coercive Control, Dating, Domestic, Gender-Based, Interpersonal, Intimate Partner, Sexual, Other.

1. Are you aware of any **measurement frameworks** of tech abuse?
2. [If yes] Please **pick your top three scales** according to your preference regarding how effectively they capture and measure tech abuse phenomena:

Cyber Dating Abuse Questionnaire (CDAQ), Cyber Dating Violence Inventory (CDVI), Cyber Psychological Abuse (CPA) Scale, Digital Dating Abuse (DDA), Partner Cyber Abuse Questionnaire (PCAQ), Sexual Image-Based Abuse Myth Acceptance Scale (SIAMA), Technology-Facilitated Abuse in Relationships (TAR) Scale, Technology-Facilitated Sexual Abuse Victimization (TFSVV) Scale, Other.

1. On a scale of 1 (strongly disagree) to 7 (strongly agree), how strongly would you agree or disagree that the following aspects should **guide our conceptualization** (i.e., definition and measurement) of tech abuse?

Relationships, gender, sex, sexual orientation, motive, intent, history, behavior, frequency, severity, type of technology, lack of consent, perception of harm, impact, legal thresholds, cultural norms, social norms.

1. On a scale of 1 (strongly disagree) to 7 (strongly agree), how strongly would you agree or disagree with the **statements** below?

Personal relationship, do not know one another (i.e., strangers), professional relationship, directed at someone, digital means, offline, distinct form of abuse.

1. On a scale of 1 (strongly disagree) to 7 (strongly agree), how strongly do you agree or disagree with **excluding certain groups** (as victims-survivors and/or perpetrators) from the conceptualization of tech abuse?

Children as perpetrators, Teenagers and adolescents as perpetrators, Groups/communities as victims-survivors, Groups/communities as perpetrators, Institutions as victims-survivors, Institutions as perpetrators.

1. On a scale of 1 (strongly disagree) to 7 (strongly agree), how strongly would you agree or disagree that the following **phenomena and behaviors** fall under your conceptualization of tech abuse (i.e., they are part of your understanding of tech abuse as you see it)?

Artificial Intelligence (AI) bias, Catfishing, Cyber/digital dating abuse, Cyberbullying, Cyberstalking, Employee monitoring/bossware, Ghosting, Grooming/online solicitation, Homicide in virtual gaming, Misinformation/disinformation campaigns, Online child sexual abuse/exploitations, Online harassment towards private individuals, Online harassment towards public figures, e.g., politicians or journalists, Online hate speech, Online misogynoir, Online misogyny, Online pornography, Online sexism, Online terrorism, Online trolling, Online workplace harassment, Rape in virtual gaming, Romance scams/romance fraud, Sexting, Technology-facilitated trafficking.

**Round 3 Main questions**

1. To achieve consensus on the **most-favored prefix**, please rank the following terms again. You may choose to put them in the same order as before, or change your response:

Cyber, Digital, Online, Technology.

1. To achieve consensus on the most favored term describing **technology’s role** in tech abuse, please rank the following items again. You may choose to put them in the same order as before, or change your response:

Assisted, Based, Enabled, Facilitated.

1. To achieve consensus on the most favored term describing **the scope of violence/abuse**, please rank the following items again. You may choose to put them in the same order as before, or change your response:

Against Women and Girls, Against Women, Coercive Control, Gender-Based, Intimate Partner.

1. On a scale of 1 (strongly disagree) to 7 (strongly agree), how strongly would you agree or disagree that the following aspects should **guide our conceptualization** (i.e., definition and measurement) of tech abuse?

Relationships, gender, sex, sexual orientation, motive, intent, history, frequency, severity, type of technology, legal thresholds, cultural norms, social norms.

1. On a scale of 1 (strongly disagree) to 7 (strongly agree), how strongly would you agree or disagree with the **statements** below?

Directed at someone, distinct form of abuse

1. On a scale of 1 (strongly disagree) to 7 (strongly agree), how strongly do you agree or disagree with **excluding certain groups** (as victims-survivors and/or perpetrators) from the conceptualization of tech abuse?

Children as perpetrators, Institutions as victims-survivors

1. On a scale of 1 (strongly disagree) to 7 (strongly agree), how strongly would you agree or disagree that the following **phenomena and behaviors** fall under your conceptualization of tech abuse (i.e., they are part of your understanding of tech abuse as you see it)?

Artificial Intelligence (AI) bias, Employee monitoring/bossware, Ghosting, Homicide in virtual gaming, Misinformation/disinformation campaigns, Online pornography, Online terrorism, Sexting.

**Appendix D. Follow-up interview questions**

**General prompts**

1. What did you **think** of our Delphi study overall?
2. How do you **feel** about the findings and the items that reached consensus?
3. What was your own personal **thought and decision process** behind selecting a particular term/definition/measurement tool? [the interviewer will have checked the participant’s responses prior to the interview]
4. Having seen the findings of the Delphi study, what’s your **“take home”** message?
5. What do you think would be the best way to **agree on and popularise** a unified/standardized terminology, definition, and measurement moving forward?

**Question-specific prompts**

1. In the question regarding the **scope of tech abuse**, we had varied ratings on whether a gender-specific element should be added to the definition and which one is the best (gender-based, against women and girls, against women, etc.). What’s your opinion on this?
2. When we asked participants if they were aware of **frameworks to measure tech abuse**, only 32.8% said that they were aware of such tools. Why do you think this is?
3. Participants also appeared conflicted as to whether **protected characteristics** (e.g., gender, sexual orientation) or **social and cultural norms** should guide our conceptualization of tech abuse. What’s your opinion on this?
4. Should tech abuse be **directed** at someone? This was another item on which there was no consensus.
5. Do you think the definition needs to follow the **legal thresholds** for what should be considered tech abuse?
6. We also asked whether **certain groups should be excluded** from the conceptualization of tech abuse. The item as to whether children should be excluded as perpetrators did not reach consensus. Where do you stand on this? [And follow-up question if relevant/necessary] In your mind, is there an age limit for the perpetrator to be considered tech abuse?
